# 1203. A Descriptive Analysis of an Opioid Use Disorder Care Continuum in an Infectious Diseases Clinic

**DOI:** 10.1093/ofid/ofab466.1395

**Published:** 2021-12-04

**Authors:** Sarah R Blevins, James A Grubbs, Tiffany Stivers, Kathryn Sabitus, Ryan Weeks, Alice Thornton

**Affiliations:** 1 University of Kentucky HealthCare, Lexington, Kentucky; 2 University of Kentucky, Lexington, Kentucky

## Abstract

**Background:**

On December 17, 2020, U.S. CDC released an advisory reporting the highest drug overdose rate on record. Kentucky ranks in the top 5 states for opioid overdose deaths. Retention in opioid use disorder (OUD) treatment is associated with decreased overdose deaths. University of Kentucky HealthCare’s infectious disease division (UKID) implemented a multi-disciplinary approach to expand access to medication for opioid use disorder (MOUD) for patients with injection drug use-associated infections (IDU-AI). This program is modelled after the Ryan White Cares Act to engage and retain patients.

**Methods:**

. This ongoing project began enrollment in June 2019. Any patient ≥18 years old with IDU-AI and OUD is eligible for enrollment unless pregnant or incarcerated. Patients are eligible for transportation assistance, mental health services, and medical case management. They may start MOUD with UKID or be referred elsewhere. In this analysis, we describe our opioid use disorder care continuum and identify reasons for patient attrition and areas to improve

**Results:**

Our continuum components are referral, eligible, enrolled, start MOUD, and retention at month 1, 3, and 6. To date, 533 patients have been referred. Of these, 383 (71.9%) were eligible and 150 (39%) enrolled. Reasons patients did not enroll: discharged stable (41.5%), left AMA (16.9%), declined (10.8%), deceased (6.7%), discharged to other hospital (3.6%), missed clinic visit (9.7%), hospice (1%), other (10.8%). Reasons patients declined: no reason (28.6%), refused to discuss (19.1%), no interest (14.3%), travel (4.8%), declined ID follow-up (4.8%), time limits (9.5%). Ninety-three patients have been enrolled ≥6 months; 83 are on MOUD. Sixty-seven, 29, and 20 patients were retained at month 1, 3, and 6, respectively.

**Conclusion:**

UKID engages patients in OUD treatment, but retention rates are comparable to those described in non-ID settings. Most attrition occurs between eligibility and month 3, suggesting patients are most vulnerable when they consider change and start MOUD. These time points should be priority for patient engagement by clinic staff. Also our staff size struggles to meet the demand. The number of referrals is prohibitive for our small team to approach everyone in a timely manner. More programs like this one are needed.

**Disclosures:**

**All Authors**: No reported disclosures

